# Blood parameters and pathological lesions in pigs experimentally infected with Vietnam's first isolated African swine fever virus

**DOI:** 10.3389/fvets.2022.978398

**Published:** 2022-09-08

**Authors:** Sang-Ik Oh, Thi Thu Huyen Nguyen, Myeon-Sik Yang, Bui Thi To Nga, Vuong Nghia Bui, Van Phan Le, Seung-Won Yi, Eunju Kim, Tai-Young Hur, Hu Suk Lee, Bumseok Kim

**Affiliations:** ^1^National Institute of Animal Science, Rural Development Administration, Wanju, South Korea; ^2^College of Veterinary Medicine, Jeonbuk National University, Iksan, South Korea; ^3^Bac Giang Agriculture and Forestry University, Hanoi, Vietnam; ^4^College of Veterinary Medicine, Vietnam National University of Agriculture, Hanoi, Vietnam; ^5^Virology Department, National Institute of Veterinary Research, Hanoi, Vietnam; ^6^International Livestock Research Institute, Hanoi, Vietnam; ^7^College of Veterinary Medicine, Chungnam National University, Daejeon, South Korea

**Keywords:** African swine fever virus, pathology, blood count, biochemical parameter, histopathology, viral load, pathobiology

## Abstract

African swine fever virus (ASFV) is a notable virus and one of the most serious global threats to the pig industry. Improving awareness about host–virus interactions could facilitate the understanding of the disease pathogenesis. Therefore, we investigated changes in blood parameters, viral loads, and pathological changes in ASFV-inoculated pigs according to the time of death after the onset of viremia. For the analyses, the ASFV-infected pigs (*n* = 10) were divided into two groups (five pigs/group) according to their time of death after the onset of viremia. The blood cell count dynamics and serum biochemistry profiles were similar between the groups; however, viral load distribution was different. A comparison of the histopathological changes and immunohistochemistry results between the two groups indicated that the lymphoid system, particularly the spleen, was more damaged in the early stage of the disease than in the last stage. Additionally, the virus-induced lesions in other organs (liver and kidney) were more severe in the late stage than in the early stage. Our findings provide invaluable information on the characteristics of blood parameters and pathological lesions in pigs infected with the Asia-epidemic ASFV strain and the course of ASF, targeting internal organs in pigs. Overall, this study characterizes the host-pathogen interaction in ASFV infection, offering insight for the establishment of ASF control strategies.

## Introduction

African swine fever (ASF) is a serious infectious disease in domestic pigs caused by the African swine fever virus (ASFV), which has a fatality rate approaching 100%. ASFV is a large, double-stranded DNA virus (170–193 kbp) that belongs to the *Asfivirus* genus of the family *Asfarviridae* (1, 2). To date, eight ASFV serotypes have been established based on the viral hemagglutinin CD2-like protein (CD2v) and C-type lectin 24 genotypes with different levels of virulence are known (2, 3). In 2007, genotype II ASFV was introduced into Georgia, which spread to neighboring European countries, finally reaching China in August 2018; the first outbreak of ASF in East and South-East Asia was reported in Liaoning (4). In February 2019, ASFV (VUNA/HY/Vietnam) was detected in a backyard pig farm in northern Vietnam, located ~250 km from the Chinese border (5). This Asian strain has the characteristics of the intergenic region II variant of genotype II, with an additional tandem-repeat sequence (5′-GGAATATATA-3′) between the 173R and 1329L genes (6–8).

Since no treatment is available for ASF, several studies have attempted to develop ASF vaccines (9–11). However, currently, there are no commercially available vaccines to prevent ASF in pigs (12, 13). Therefore, the control of ASF outbreaks depends on rapid and accurate diagnosis by veterinarians and monitoring of healthy pigs (14). ASFV-infected pigs usually develop viremia at 4–8 days post-inoculation (dpi). Considering the absence of fully neutralizing antibodies, viremia could persist for weeks or months (15). The lymph node macrophages and monocytes nearest the point of entry are the primary cells for ASFV replication (15, 16). The replicated virus spreads through the blood and lymphatic system to all organs, including the spleen, other lymph nodes, tonsils, lungs, liver, and kidneys (15). Subsequently, ASFV-infected domestic pigs develop severe clinical signs (e.g., fever, cough, difficulty breathing, decreased appetite, and hemorrhagic skin, and finally die within 7–13 days, with various histopathological features, including splenomegaly, hemorrhagic lymph nodes, severe pulmonary edema, and petechial hemorrhage in the kidneys (17, 18).

Understanding ASF pathogenesis may help predict the course of the disease, laying the groundwork for developing vaccines and therapies (15). Understanding pathology is crucial for understanding pathogenesis and may complement other approaches to better understand host–virus interactions (17). Pathological lesions and clinical signs in ASFV-infected pigs vary depending on the virulence of the virus strains, host characteristics, dose, and route of infection (19). Several studies have reported the clinical signs, pathological changes, and blood parameters in pigs experimentally infected with European strains of ASFV (20–22). However, there have been few pathophysiological studies on the comprehensive changes in pigs which inoculated with the Asian-epidemic strain (4). In Vietnam, some studies and case reports on the molecular profile, pathogenicity, transmission, and pathology of ASF have been published (8, 23–25), including our study that showed that the VUNA/HY/Vietnam strain in the peracute/acute phase led to the death of pigs at 7.0 ± 1.2 dpi, with a short incubation time (3.7 ± 0.5 dpi) (24). However, these results are insufficient to understand the host–virus interactions of the Vietnamese strain.

To better understand these host–virus interactions, we investigated changes in the blood parameters, viral loads, and pathological lesions in pigs experimentally infected with the VUNA/HY/Vietnam ASFV strain, which were grouped according to the time of death after the onset of viremia. The findings provide invaluable information on the biochemical parameters and pathological lesions in pigs experimentally infected with the Asian ASFV strain (peracute to acute form) and thus will help determine the course of ASF in the internal organs of domestic pigs.

## Materials and methods

### Virus strain

In this study, domestic pigs were experimentally infected with ASFV, VNUA/HY/Vietnam strain (GenBank accession number: MK554698). The virus was isolated from the spleens of naturally infected pigs during the first outbreak in Hung Yen Province, north of Hanoi, Vietnam, in February 2019 (5). The virus was propagated in porcine alveolar macrophages using Dulbecco's modified Eagle's medium supplemented with 5% fetal bovine serum and stored at −80°C. A hemadsorption assay was used to titrate the virus, as previously described (26). The isolated virus was added to 96-well plates containing primary porcine alveolar cells in triplicate. Ten-fold serial dilutions were performed. The 50% hemadsorbing dose per milliliter (HAD_50_/ml) was calculated for 7 dpi. Virus titers were calculated using the method described by Reed and Muench (27).

### Experimental design

We used 15 healthy 7–8-week-old pigs (Yorkshire × Landrace × Duroc) obtained from two sows in the same herd at a commercial pig farm. All pigs were confirmed to be seronegative for the endemic pathogens in pigs from Vietnam: ASFV, porcine circovirus 2, foot-and-mouth disease, classical swine fever virus, and porcine reproductive and respiratory syndrome virus. All pigs were carefully monitored daily for seven days before ASFV inoculation. The detailed experimental procedure has been described previously (24). Ten pigs were intramuscularly inoculated with 1 ml of ASFV at a titer of 10^3.5^ HAD_50_/ml per pig (ASFV-infected group), and five pigs were not inoculated (negative control group). The ASFV-infected group was assigned to an animal biosafety level 2 facility at the National Institute of Veterinary Research, Hanoi, Vietnam. The study was conducted according to the guidelines of the National Institute of Veterinary Research, Vietnam, and approved by the Institutional Animal Care and Use Committee of the National Institute of Animal Science, Republic of Korea (approval number: NIAS 2020-463). Whole blood was collected daily from the 10 ASFV-infected pigs after experimental inoculation with ASFV. DNA was extracted, and the presence of ASFV was assessed using a VDx ASFV qPCR kit (Median Diagnostics, Chuncheon, Korea) following the manufacturer's instructions. The onset of viremia in each pig was determined as the day when the cycle threshold value of the collected blood sample was <40. ASFV-infected pigs were divided into two groups according to the time of natural death after the onset of viremia. Group I contained five pigs that died 2–5 days post-viremia (dpv), and Group II comprised five additional pigs that died at 6–7 dpv.

### Blood count analysis

Blood samples were collected daily from the jugular vein of each pig before feeding in the morning. Blood samples were placed into BD Vacutainer K2 ethylene diamine tetra-acetic acid (EDTA) tubes (BD Biosciences, Franklin Lakes, NJ, USA) containing 2 ml anticoagulant solution (EDTA). An automated hematology analyzer (Mindray BC-2800 Vet; Mindray Bio-Medical Electronics Co., Ltd., Shenzhen, China) was used to perform a complete blood cell count [white blood cells (WBCs), red blood cells (RBCs), hemoglobin levels, and platelet counts] (22). Results were compared to the calibrated Mindray references for pigs.

### Serum and biochemical parameters

Blood samples were collected daily from the jugular vein of each pig before feeding in the morning and placed in plain (5 ml) BD Vacutainer tubes (BD Biosciences, Franklin Lakes, NJ, USA). Serum was separated by centrifugation at 1,800 × *g* for 10 min and stored at −20°C. Biochemical analyses were performed using a semi-automatic biochemical analyzer (Mindray BA-88A; Mindray, Shenzhen, China). Measurements of aspartate aminotransferase (AST), alanine aminotransferase (ALT), creatinine, and urea were used to diagnose organ damage, especially to the liver and kidneys. All procedures for the analysis of biochemical parameters were performed at the Viet Pet Clinic in Hanoi, Vietnam.

### Gross pathology and tissue collection

Complete necropsies were performed on all pigs according to the standardized macroscopic lesion guidelines for ASFV infection in pigs (28). Gross lesions were observed in ten organs, including the spleen; submandibular, mesenteric, and inguinal lymph nodes; liver, lungs, kidneys, tonsils, heart, and colon. During necropsy, tissue samples of the ten organs were collected from four pigs according to their time of death [Group I (*n* = 2, died at 3–4 dpv); Group II (*n* = 2, died at 6–7 dpv)].

### Viremia and viral gene (p72) detection in tissue samples

To detect viremia, DNA was extracted from whole blood using a nucleic acid extraction kit (DNeasy Blood & Tissue Kit; Qiagen, Hilden, Germany). A total of 100 tissue samples (~1 g each) from ten necropsied pigs were homogenized in 3 ml of sterile phosphate-buffered saline. DNA was extracted from 100 μl aliquots of each homogenized tissue sample according to manufacturer instructions (DNA Mini Kit; Qiagen, Hilden, Germany). The extracted DNA was analyzed for the presence of ASFV DNA using a VDx ASFV qPCR kit (Median Diagnostics). Briefly, 5 μl DNA was placed in a tube containing 10 μl 2× master mix and 5 μl 4× oligo mix. A quantitative polymerase chain reaction was performed using the IQ5 Multicolor Real-Time PCR Detection System (Bio-Rad Laboratories Ltd., Hercules, CA, USA). The reaction conditions used have been described previously (24). Blood samples with a cycle threshold value of <40 were considered positive for ASFV. Copy numbers were calculated based on the standard samples provided by the manufacturer.

### Histopathology and immunohistochemistry

Tissue samples (spleen; submandibular, mesenteric, and inguinal lymph nodes; liver; lungs; kidneys; tonsils; heart; and colon) were collected from four pigs (#54, #48, #60, and #43) among the experimental 10 pigs and immersed in 10% neutral buffered formalin. Pigs #54 and #48 were included in Group I, and pigs #60 and #43 were in Group II. Representative sections of each tissue sample were cut and embedded in paraffin. Paraffin-embedded tissues were sectioned at a thickness of 3–4 μm and then subjected to hematoxylin and eosin staining for histopathological examination. Histopathological changes were categorized according to the protocols of Galindo-Cardiel et al. (28) and Sehl et al. (21), as follows: normal (0), mild (1), moderate (2), and severe (3).

For ASFV immunohistochemistry, a monoclonal antibody against the p72 major capsid protein (clone 1BC11; Ingensa, Madrid, Spain) was used as the primary antibody. The Simple Stain MAX PO method with Histofine Simple Stain MAX PO (MULTI; Nichirei Biosciences, Tokyo, Japan) was used as the secondary antibody. Briefly, tissue sections were deparaffinized and immersed in 3% hydrogen peroxide and methanol to block endogenous peroxidase. The tissue sections were rinsed with Tris-buffered saline containing 5% Tween 20 (TBS-T) before antigen retrieval by heating the slides in citrate buffer (pH 6.0; 0.05% Tween 20) at 120°C for 10 min. The tissue sections were incubated with 10% normal goat serum at 25°C for 15 min prior to primary antibody incubation overnight at 4°C. The primary antibody was diluted 1:300 in TBS containing 1% bovine serum albumin. As negative controls, duplicate sections were incubated with 1% bovine serum albumin in TBS instead of the primary antibody. The tissue sections were thoroughly rinsed with TBS-T and incubated with the secondary antibody, Universal Immuno-enzyme Polymer (Simple Stain MAX PO), for 30 min. After rinsing the tissue sections with TBS-T, 3,3′-diaminobenzidine (Dako, Tokyo, Japan) was used as the chromogen. Finally, the tissue sections were rinsed with tap water and counterstained with Mayer's hematoxylin. ASFV antigen detection using the immunohistochemical method was scored according to the proportion of positively stained mononuclear cells or macrophages in three fields under ×400 magnification, as follows: no positive cells (0), 1–10 positive cells (1), 11–20 positive cells (2), 21–30 positive cells (3), 31–40 positive cells (4), and ≥ 41 positive cells (5).

### Statistical analyses

All statistical analyses were conducted using SPSS (version 26.0; IBM Corp., Armonk, NY, USA). Biochemical parameters and mean viral copies in blood for Group I, Group II, and the negative control group were analyzed using one-way analysis of variance and Duncan's test. The viral load in the 10 collected tissue samples was analyzed by Student's *t*-test between Groups I and II. A *p*-value < 0.05 was considered statistically significant.

## Results

### Viral detection in blood

**Figure 1:**
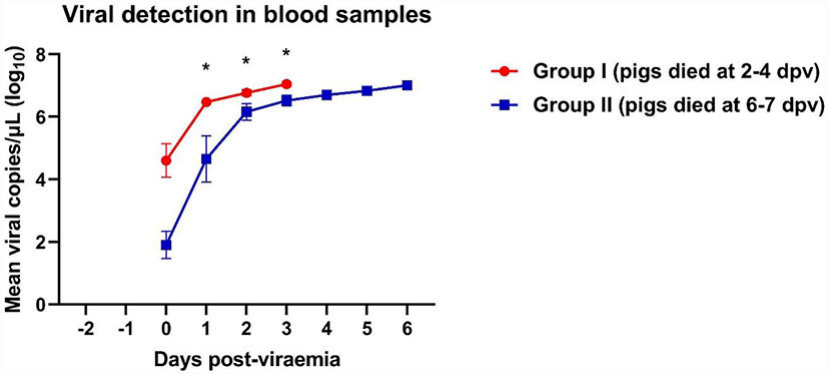
Viral load in pigs experimentally infected with African swine fever virus. Mean viral copy number per microliter in two groups divided by the number of days between the onset of viremia and death. *p < 0.05. dpv, days post-viremia.

A rapid increase in virus titers was observed in both groups of ASFV-infected pigs (Figure 1). Notably, Group I (died at 2–5 dpv) pigs had an ~10^3^-fold higher copy number of the ASFV than Group II (died at 6–7 dpv) pigs (8.5 × 10^5^ ± 8.2 × 10^5^ vs. 7.0 × 10^2^ ± 6.5 × 10^2^ copies/μl, respectively; *p* = 0.336). Virus titers markedly increased 1 day after the onset of viremia. Pigs started dying when the copy number exceeded 4.3 × 10^6^ copies/μl in Group I and 6.0 × 10^6^ copies/μl in Group II. The mean viral load in the blood of dead pigs in Group I was significantly higher than that in the blood of dead pigs in Group II at 1 dpv (*p* = 0.011), 2 dpv (*p* = 0.032), and 3 dpv (*p* = 0.021). The mean ASFV copy number dramatically increased between 0 (8.5 × 10^8^ ± 6.9 × 10^8^ copies/μl) and 1 dpv (3.3 × 10^6^ ± 5.2 × 10^8^ copies/μl) in Group I, and between 0 dpv (6.9 × 10^2^ ± 6.5 × 10^2^ copies/μl) and 2 dpv (2.3 × 10^6^ ± 7.5 × 10^8^ copies/μl) in Group II. The viral load subsequently increased until all pigs died [Group I: 5 dpv (1.1 × 10^7^ copies/μl); Group II: 6 dpv (1.1 × 10^7^ ± 2.4 × 10^6^ copies/μl)]. The detailed results of the viral detection in blood samples from each pig are shown in Supplementary Table 1.

### Clinical sign score

**Figure 2:**
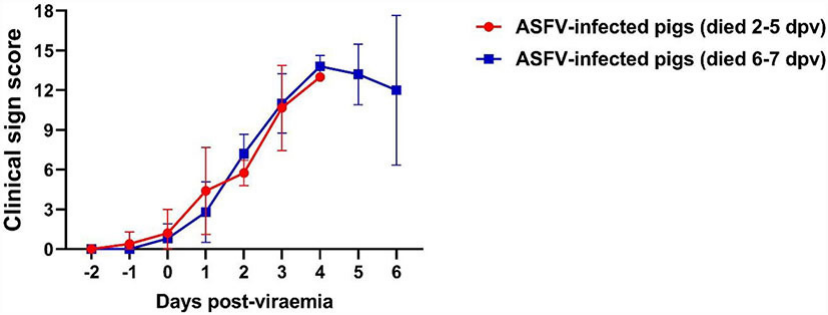
Clinical sign scores in two groups. The red line (Group I) represents the pigs that died at 2–5 days post-viremia (dpv), and the blue line (Group II) represents the pigs that died at 6–7 dpv.

The clinical signs exhibited by each pig were scored according to a method described in our previous study (24). The average onset time of clinical symptoms (over 3 scores) was 1.4 ± 0.6 dpv (Group I: 1.2 ± 0.7 dpv; Group II: 1.6 ± 0.5 dpv). The dynamics of the clinical sign scores in both groups were similar (Figure 2).

### Blood counts

**Figure 3:**
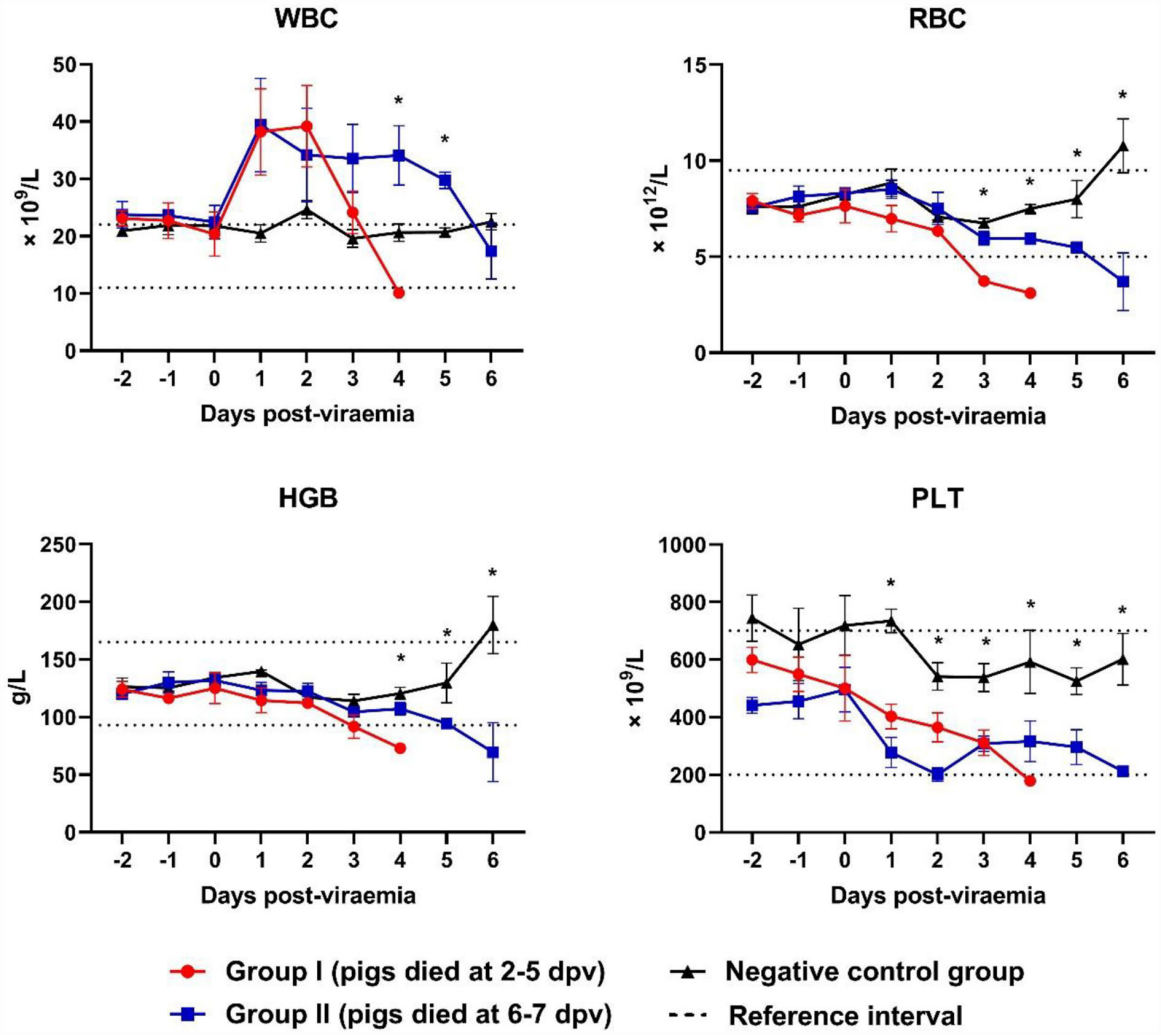
Blood counts during infection. *p < 0.05. WBC, white blood cell, RBC, red blood cell; HGB, hemoglobin; PLT, platelet. The reference intervals of each blood parameter are represented as dotted lines.

White blood cell (WBC) counts varied according to the stage of ASFV infection. WBC counts rapidly increased at 0–1 dpv in both ASFV-infected groups and sharply declined at 2–4 dpv in Group I and 4–6 dpv in Group II (Figure 3). Leukocytosis occurred mainly during the first few days after the onset of viremia in eight ASFV-infected pigs [Group I (4/5) and Group II (4/5)]. Leukocytopenia was observed during the last few days of life in one pig in Group I; however, a tendency toward leucopenia was notable in Groups I and II. The WBC count in Group II was significantly higher than that in the control group at 4 dpv (*p* = 0.040) and 5 dpv (*p* = 0.001). Erythrocytopenia was observed during the last few days of life in five pigs [Group I (4/5) and Group II (2/5)]. The lowest number of red blood cells (RBCs) was detected in pig #40 in Group I (3.1 × 10^12^ RBCs/L on the last day of life), which also presented with erythrocytopenia one day before death (3.6 × 10^12^ RBCs/L). A significant decrease in RBC count was recorded at 3 dpv (*p* < 0.001) in Group I and at 4 dpv (*p* < 0.001), 5 dpv (*p* = 0.037), and 6 dpv (*p* = 0.035) in Group II, compared with the control group. A strong tendency toward reduction in hemoglobin levels was identified. Four pigs [Group I (2/5) and Group II (2/5)] developed anemia during the last few days of life. There was a significant difference between Group II and the control group at 4 dpv (*p* = 0.020). The analysis performed 6 days after the onset of viremia showed a marked tendency toward decreased platelet count in the two ASFV-infected groups compared with that in the control group. Only two pigs presented thrombocytopenia before they died [Group I (1/5) and Group II (1/5)]. The platelet count was significantly lower in Group I than in the control group at 1 dpv (*p* < 0.001), 2 dpv (*p* < 0.001), and 3 dpv (*p* = 0.003), and in Group II than in the control group at 2 dpv (*p* < 0.001), 3 dpv (*p* = 0.003), 5 (*p* = 0.018), and 6 dpv *(p* = 0.048).

### Biochemical parameters

**Figure 4:**
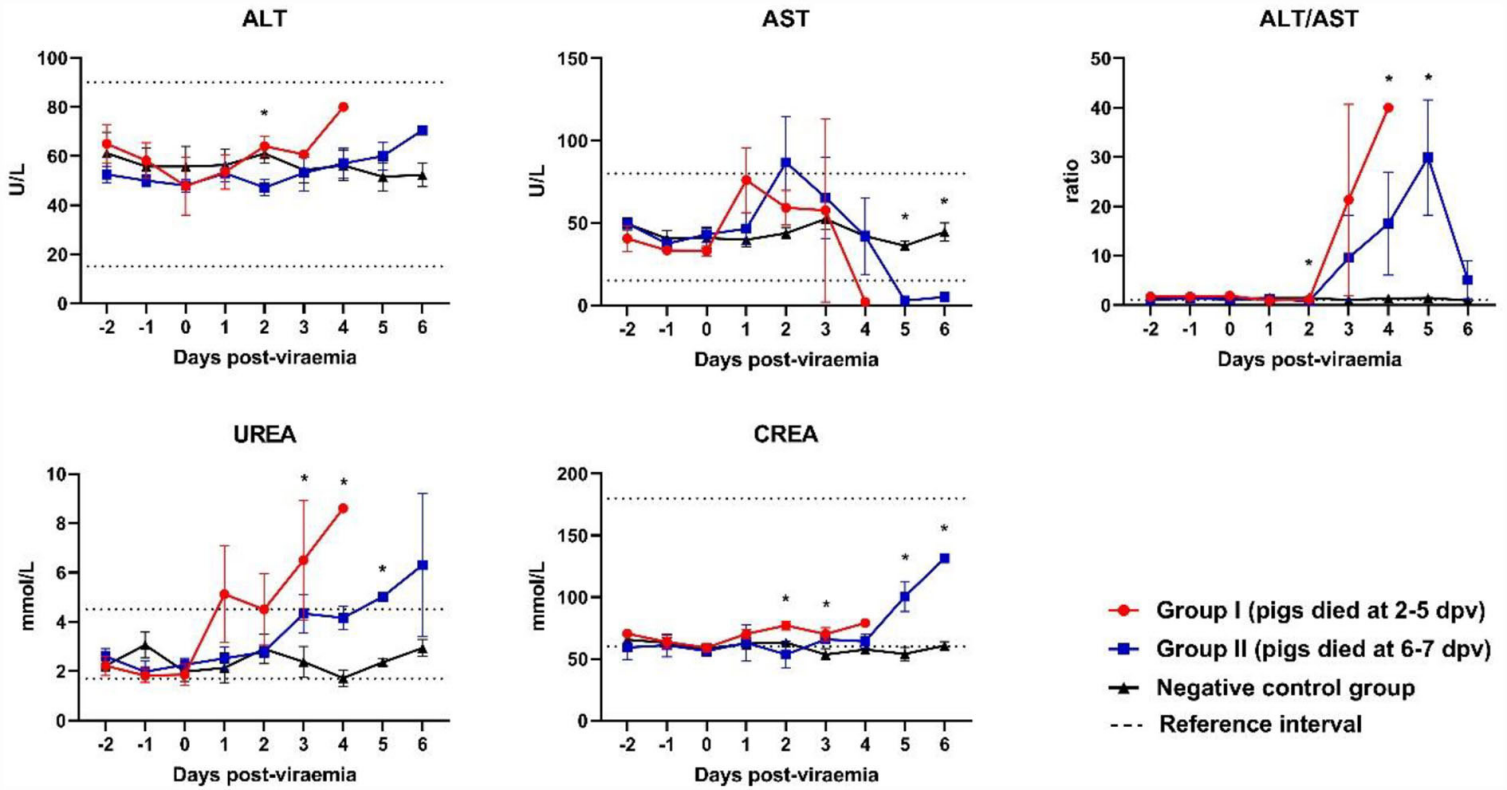
Biochemical parameters of the liver and kidneys. *p < 0.05. ALT, alanine aminotransferase; AST, aspartate aminotransferase; CREA, creatinine; UREA, urea. The reference intervals of each blood parameter are represented as dotted lines.

Although the ALT levels of all ASFV-infected pigs were included in the reference range, ALT levels gradually increased after 2 dpv (Figure 4). ALT levels in Group I pigs significantly differed from those in Group II and control pigs. A different pattern was observed in the levels of AST. AST levels peaked shortly after the onset of viremia (1 and 2 dpv in Groups I and II, respectively), followed by a reduction (below the reference range) during the last few days of life [Group I (2/5) and Group II (5/5)]. AST levels were significantly lower in Group II than in the control group at 5 dpv (*p* < 0.001) and 6 dpv (*p* = 0.008). The ALT/AST ratio dramatically increased in both ASFV-infected groups at 2 dpv. ALT/AST ratios in the ASFV-infected groups were significantly higher than that in the control group at 4 dpv (*p* = 0.013) and 5 dpv (*p* = 0.040).

A strong tendency toward increased creatinine levels was observed in Group II pigs during the last few days of life. The creatinine levels in Group I were significantly higher than those in Group II and the control group at 3 dpv (*p* = 0.029). The creatinine levels in Group II were significantly higher than those in the control group at 5 dpv (*p* = 0.008) and 6 dpv (*p* < 0.001). High urea levels (above the reference range) were observed in Group I pigs on the last day of life. Although urea levels were included in the reference range, they tended to increase in ASFV-infected pigs after the onset of viremia.

### Gross lesions

Post-mortem examinations were performed on all pigs according to a previous study (26). ASFV-infected pigs had hemorrhagic lymphadenitis in the submandibular, mesenteric, and inguinal lymph nodes (Table 1). Although splenomegaly was common in both ASFV-infected groups, Group I pigs had more severe lesions (necrosis and hemorrhage) in the spleen than Group II pigs, whereas more macroscopic lesions were observed in the various organs of Group II pigs than in the tissues of Group I pigs: pericardial fluid [Group I (2/5) and Group II (5/5)], severe pneumonia [Group II only (1/5)], petechial hemorrhages in the kidneys [Group II only (1/5)], and hemorrhage in the colon [Group I (1/5) and Group II (2/5)].

**Table 1:** Gross organ lesions.

**Organ**	**Gross lesion^†^**	**Number of pigs (%)^‡^**		
		**Group I**	**Group II**	**Control**
Spleen	Swelling	2/5 (40%)	5/5 (100%)	0/5 (0%)
	Congestion	4/5 (80%)	4/5 (80%)	0/5 (0%)
	Hemorrhage	1/5 (20%)	0/5 (0%)	0/5 (0%)
	Necrosis	1/5 (20%)	0/5 (0%)	0/5 (0%)
Lymph nodes^§^	Swelling	5/5 (100%)	5/5 (100%)	0/5 (0%)
	Hemorrhage	5/5 (100%)	5/5 (100%)	0/5 (0%)
Tonsils	Swelling	5/5 (100%)	5/5 (100%)	0/5 (0%)
	Hemorrhage	5/5 (100%)	5/5 (100%)	0/5 (0%)
Liver	Swelling	5/5 (100%)	5/5 (100%)	0/5 (0%)
	Hemorrhage	1/5 (20%)	0/5 (0%)	0/5 (0%)
Lungs	Swelling	4/5 (80%)	4/5 (80%)	0/5 (0%)
	Hemorrhage	4/5 (80%)	4/5 (80%)	0/5 (0%)
	Pneumonia	0/5 (0%)	1/5 (20%)	0/5 (0%)
Kidneys	Swelling	4/5 (80%)	4/5 (80%)	0/5 (0%)
	Hemorrhage	0/5 (0%)	1/5 (20%)	0/5 (0%)
Heart	Pericardial fluid	2/5 (40%)	5/5 (100%)	0/5 (0%)
Colon	Hemorrhage	1/5 (20%)	2/5 (40%)	0/5 (0%)

† Macroscopic lesions were analyzed according to a previously published guideline (26).

‡ Group I, ASFV-inoculated pigs (died 2–5 dpv); Group II, ASFV-inoculated pigs (died 6–7 dpv); Control, negative control group (not inoculated with ASFV).

§ Submandibular, mesenteric, and inguinal lymph nodes.

ASFV, African swine fever virus; dpv, days post-viremia.

### Organ viral loads

**Figure 5:**
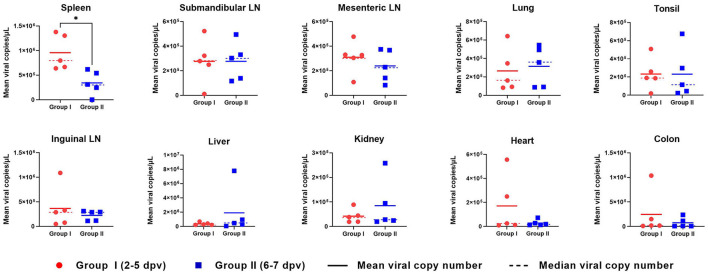
Organ viral load in pigs experimentally infected with African swine fever virus. The mean (solid lines) and median (dotted lines) viral copy number per microliter in two groups divided by the number of days between the onset of viremia and death are shown. *p < 0.05. dpv, days post-viremia; LN, lymph node.

ASFV DNA was detected in all 10 organs evaluated in ASFV-infected pigs (Figure 5). In the spleen, the mean viral load was significantly higher in Group I pigs than in Group II pigs (9.6 × 10^5^ ± 1.3 × 10^5^ *vs*. 3.4 × 10^5^ ± 9.0 × 10^4^ copies/μl, respectively; *p* = 0.013). Moreover, the mean viral load in the spleen tended to decrease from the early to the late stage of infection (death at 2, 3, 4, 5, 6, and 7 dpv: 1.4 × 10^6^, 1.3 × 10^6^, 7.2 × 10^5^, 6.7 × 10^5^, 3.7 × 10^5^, and 3.1 × 10^5^ copies/μl, respectively). Detailed information for each pig regarding the onset of viral detection in blood and time of death is shown in Supplementary Table 1. The mean viral load in submandibular and mesenteric lymph nodes, lung, and tonsil exhibited minimal difference between the two groups. Although the mean values from the inguinal lymph node, liver, kidney, heart, and colon between pigs from Group I and II were different, which could have been caused by one or two pigs having particularly high virus titers, the median values of viral titer in the spleen, mesenteric lymph node, and tonsil were higher in Group I than in II, whereas those in the lung were higher in Group II than in Group I. Overall, according to the analyses based on median viral titer values, there were no differences between the two groups.

### Histopathology

**Figure 6:**
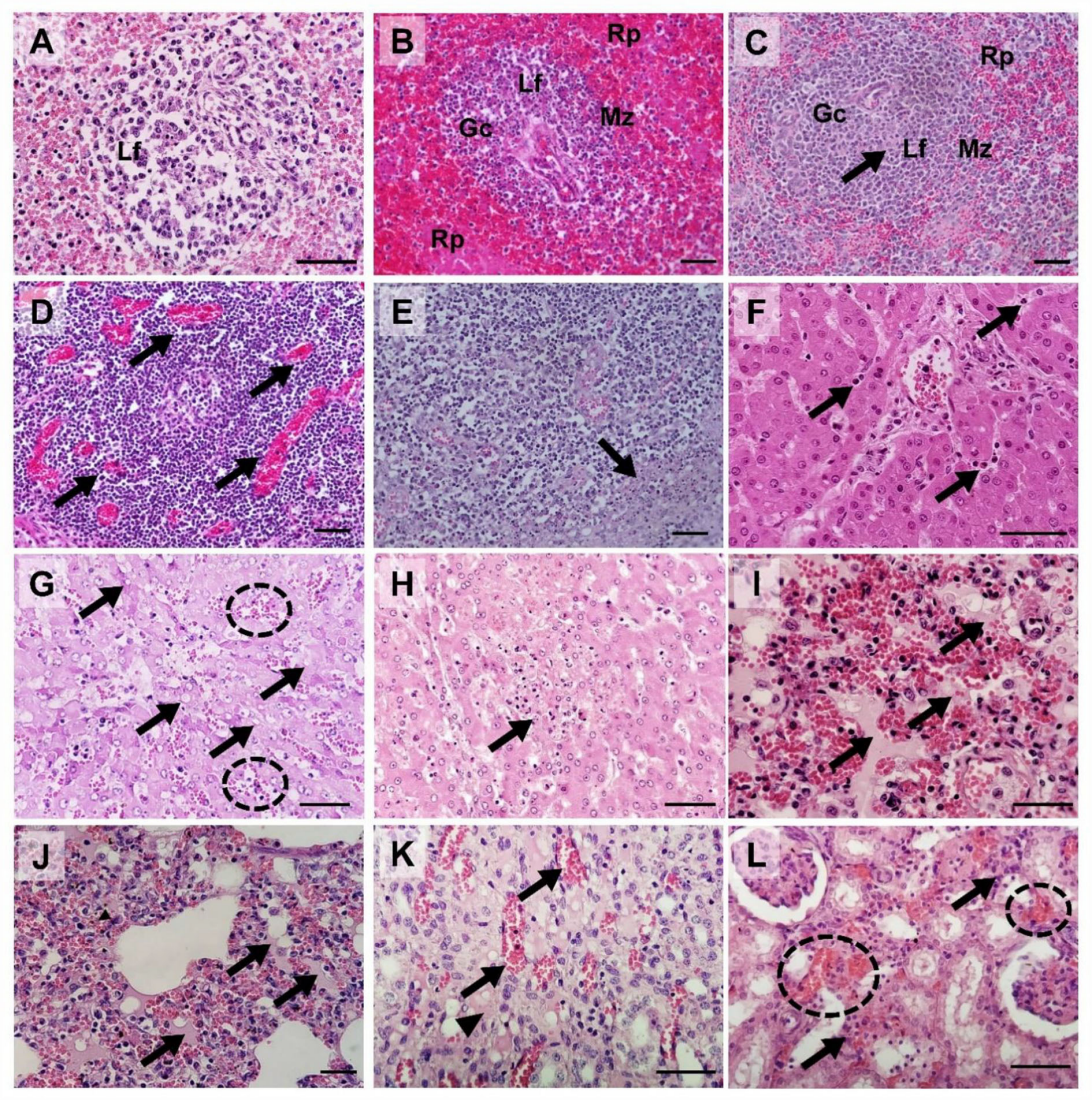
Histopathological findings in pigs experimentally infected with African swine fever virus. **(A)** Spleen [pig #54: died at 3 dpv [(Group I)]: Severe lymphoid depletion with the presence of pyknosis and karyorrhexis in the lymphoid follicle (Lf); **(B)** Spleen [pig #60: died at 6 dpv (Group II)]: Mild lymphocytic depletion in germinal center (Gc), The marginal zone (Mz) infiltrated by erythrocytes, severe and diffuse engorgement in the red pulp (Rp); **(C)** Spleen [pig #48: died at 4 dpv (Group I)]: Necrosis of lymphocytes (arrow) within the lymphoid follicle (Lf); **(D)** Submandibular lymph nodes (pig #54): Severe congestion (arrows); **(E)** Submandibular lymph nodes (pig #60): Focal necrosis (arrow); **(F)** Liver (pig #54): Neutrophils (arrows) in sinusoids. **(G)** Liver (pig #60): Multifocal congestions (dash circle) and the cytoplasmic vacuoles (arrows); **(H)** Liver (pig #43: died at 7 dpv [Group II]): Vacuolar degeneration with necrotic hepatocytes (arrow); **(I)** Lungs (pig #54): Pulmonary hemorrhages with edema (arrows); **(J)** Lungs (pig #60): Moderate pulmonary hemorrhages with edema fluids (arrows) and interstitial pneumonia; **(K)** Kidney (pig #48): Congestion (arrows) and interstitial edema (arrowhead) in the renal medulla.; **(L)** Kidney (pig #60): Multifocal hemorrhage (dash circles) and mononuclear cell infiltration in the renal interstitium (arrows). Hematoxylin and eosin stain (×400 magnification; scale bar, 50 μm). dpv, days post-viremia; Gc, germinal center; Lf, lymphoid follicle; Mz, marginal zone; Rp, red pulp.

Hematoxylin and eosin-stained spleen; submandibular, mesenteric, and inguinal lymph node; liver, lung, kidney, tonsil, heart, and colon sections from four ASFV-infected pigs [Group I (#54 and #48) and Group II (#60 and #43)] were examined and scored semi-quantitatively based on previous reports (21, 28). Supplementary Table 2 presents the histopathological lesion scores for the four pigs. Figure 6 presents the histopathological findings of various tissues in the ASFV-infected pigs in this study.

Mild-to-severe vascular damage was observed in the spleen of all necropsied pigs. In Group I pigs, moderate lymphocytolytic lesions were present; however, in Group II pigs, the lesions were mild. Lymphoid depletion was moderate-to-severe in Group I, whereas it was mild-to-moderate in Group II pigs. Necrosis of lymphocytes was moderate-to-severe in Group I pigs and mild in Group II pigs.

Moderate-to-severe vascular congestion was also observed in the submandibular lymph nodes of Group I pigs, whereas mild congestion was observed in Group II pigs. Lymphocyte apoptosis (lymphocytolysis) was detected in the lymphoid follicles of one pig in Group I (#54) that died soon after the onset of viremia. Mild-to-moderate necrosis of endothelial cells was detected in Group I pigs, whereas severe necrotic lesions were observed in Group II pigs.

In the liver, diffuse congestion and hemorrhage were observed in one pig in Group I (#48) and both pigs in Group II (#60 and #43). In pig #54, which died at 3 dpv, mild congestion and hemorrhage were observed in the absence of hepatocyte necrosis. Histopathology showed lymphoid infiltrates, mainly in the hepatic sinus. The extent of the lesion gradually increased from mild (pig #54, which died at 3 dpv) to severe (pig #43, which died 7 at dpv).

Histopathological analysis of the lungs showed moderate-to-severe pulmonary congestion in all necropsied pigs in this study. Hemorrhagic lesions were observed in one pig in each of the two groups (#54 and #60, respectively). These pigs also exhibited moderate-to-severe pulmonary edema and interstitial lung inflammation. Mild alveolar edema and interstitial pneumonia were detected in pigs without lung hemorrhagic lesions (#48 and #43).

Hemorrhagic lesions were observed in only one pig by examining gross renal lesions. However, histopathological examination detected mild-to-severe kidney vasculopathy in all ASFV-infected pigs. Group II pigs also had moderate-to-severe interstitial nephritis, which was not observed in Group I pigs. Accordingly, the mean total histopathological score in Group II was considerably higher than that in Group I (8.0 vs. 3.5, respectively).

### Immunohistochemistry

**Figure 7:**
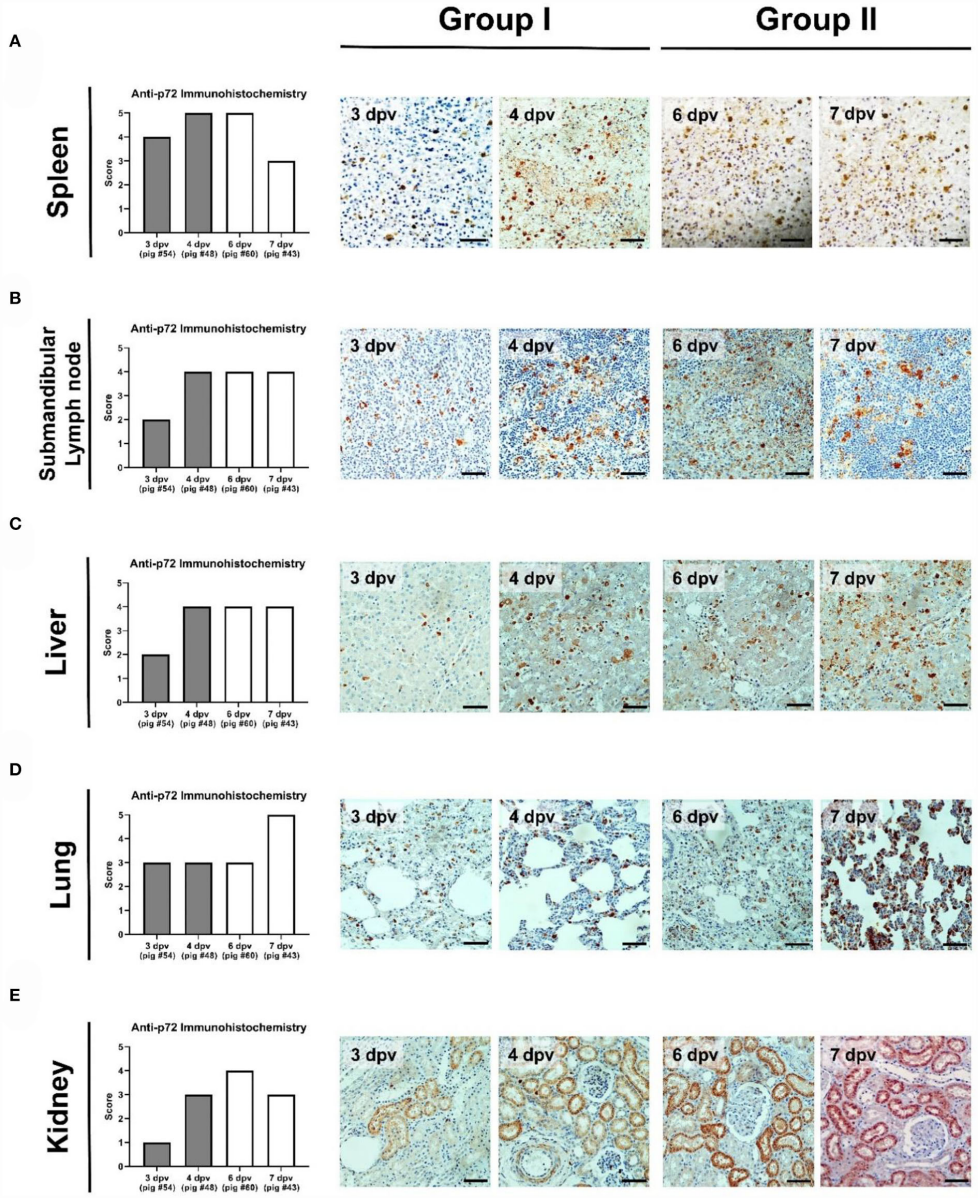
Immunohistochemical analysis of p72 in the organs of pigs experimentally infected with African swine fever virus. **(A)** Spleen, **(B)** submandibular lymph nodes, **(C)** liver, **(D)** lungs, and **(E)** kidneys scored according to the proportion of positively stained mononuclear cells or macrophages in three fields under ×400 magnification. No positive cells (0), 1–10 positive cells (1), 11–20 positive cells (2), 21–30 positive cells (3), 31–40 positive cells (4), ≥41 positive cells (5). Scale bar, 50 μm.

The immunohistochemical analysis detected numerous p72-positive cells in the spleens of three pigs that died at 3 dpv (#54), 4 dpv (#48), and 6 dpv (#43), respectively (score, 4–5; Figure 7A). A relative reduction was observed in pig #43, which died at 7 dpv (score 3). Compared to the other three pigs, pig #54, which died at 3 dpv, had fewer p72-positive cells in the submandibular lymph nodes. A higher proportion of ASFV antigen-positive cells was observed in the submandibular lymph nodes of Group II pigs (score 4; Figure 7B). Few p72-positive cells were observed in the liver of pig #54 (score 2). Conversely, abundant p72-positive cells were observed in the livers of the other three pigs (score 4; Figure 7C). In the lungs, viral antigens were detected multifocally in the mononuclear cells of all ASFV-infected pigs (score ≥3; Figure 7D). A particularly high proportion of p72-positive cells was detected in the lungs of pig #43, which died at 7 dpv (score 5). In the kidneys, the proportion of p72-positive cells was smaller in Group I (average score 2) than in Group II (average score 3.5), which exhibited a similar trend to that in the histopathological analysis (Figure 7E). The immunohistochemical scores of the mesenteric and inguinal lymph nodes, tonsils, heart, and colon of ASFV-infected pigs are shown in Supplementary Table 3.

## Discussion

The diagnosis of ASF in pigs may be complicated if pigs are infected with an ASFV strain that has not yet been characterized. Moreover, ASFV can cause death in the host within a short period after infection. Therefore, a complicated diagnosis can lead to delayed identification of a novel ASF outbreak. After experimentally infecting pigs with the highly virulent VNUA/HY/Vietnam ASFV strain, we analyzed blood counts and serum chemistry in blood samples from the inoculated pigs. In addition, we observed the pathological changes and host–virus interactions in pigs that died naturally of viral infection.

Numerous studies have shown that the development of clinical signs and the onset of death after ASFV infection depend on the virulence of the virus strain, the dose of the virus, routes of infection, and individual predisposition of pigs (11, 29–31). In this study, we determined the onset of viremia and viral loads in pigs infected with the same ASFV strain (VNUA/HY/Vietnam) using identical doses and routes of infection. A recent study revealed that the 50% pig lethal dose (PLD_50_) was 1 ml of ASFV at a titer of 10^1.7^ HAD_50_/ml. Hence, a dose of 1.0 × 10^3.5^ HAD_50_/ml virus (1 ml) was used in this study to observe a sufficient pathophysiological changes by ASFV infection in the virus inoculated pigs (32). In addition, a frequently selected age (7–8-week-old) of animal for ASFV inoculation experiment was chosen to compare the pathophysiological changes between pigs infected with Vietnam's first isolated ASFV and those with virus from other countries (11, 32–34). Ten ASFV-infected pigs were divided into two groups, according to the time of natural death after the onset of viremia. Interestingly, Group I pigs, which died at 2–5 dpv, had a higher viral load in the blood at the onset of viremia than Group II pigs, which died at 6–7 dpv. These results were similar to those of previous studies which reported that pigs infected with a higher dose of ASFV showed a higher viral load in the blood and earlier time of death than those infected with a lower dose (32, 35). Our study also showed that Group I pigs had a markedly higher viral load in the blood at 1–3 dpv, compared to Group II pigs. Although all pigs in this study were inoculated with same dose of ASFV, a different viral load in the blood was observed in each individual pig at the initial time of infection. The discrepancy of the viral susceptibility in each pig may explain these findings, because the severity of ASFV is known to differ based on the individual predisposition of the host (29, 30). Overall, these findings suggest that the initial viral load in the blood of ASFV-infected pigs is an important factor in determining the severity of the clinical course and time of natural death. In addition, each pig had a distinct level of susceptibility to ASFV infection, which implied that the timing of natural death would vary among individuals even though the pigs were infected with the same amount of virus. Further genetic and immunological studies of ASFV-infected pigs are needed to elucidate the dynamics of virus susceptibility.

Macrophages and monocytes are the primary target cells for the replication of ASFV. Therefore, the virus induces the activation and destruction of monocytes and macrophages (36). The infected monocyte–macrophage system secretes proinflammatory cytokines such as interleukin-1/6 and tumor necrosis factor-alpha, which strongly induce lymphocyte apoptosis (37). Severe leucopenia is considered the result of the abovementioned immunological processes (22, 36, 37). Recent studies of European strains of ASFV showed that WBC counts in ASFV-infected pigs gradually decreased after the onset of viremia (22, 35). However, a sharp increase in WBC counts was observed in this study early after the onset of viremia (until 1–2 dpv) in both groups, resulting in leukocytosis in ASFV-infected pigs up until the last day of life. A previous study of an atypical strain from Armenia in 2011 reported that this strain (a more chronic form) showed moderate leukocytosis with a slight left shift (38). Hühr et al. (39), in contrast, reported that leukocytosis was observed in ASFV-infected wild boars but was not detected in domestic pigs. These results suggest that, compared to the European strains, the Vietnamese strain exhibits different patterns of changes in WBC counts (inflammatory processes). ASFV infection in pigs also increases vascular permeability, leading to hemorrhage and organ devastation (36). This may result in a reduction in RBC counts and hemoglobin levels, which are consistent with the findings of this study. Erythrocytopenia was observed in all ASFV-infected pigs (Groups I and II) from 2 days before death, similar to the results of a previous study on pigs infected with high-dose ASFV from Poland (22). The platelet count was significantly lower in the two ASFV-infected groups than in the control group and markedly decreased after the onset of viremia. These results were concordant with those of previous studies of other European strains (22, 35). The tendency toward thrombocytopenia may be caused by disseminated intravascular coagulation, as well as the apoptosis of megakaryocytes (36).

Serum biochemical profiles were examined to determine ASFV-induced damage to internal organs (especially the liver and kidneys) and to determine the clinical course of ASF. In the case of ALT, a marked increase was observed in all ASFV-infected pigs during the last 2–3 days of life, which is consistent with the findings of a previous study by Semerjyan et al. (40) on the Georgia strain in 2007. We also observed a marked increase in AST levels [Group I (1 dpv) and Group II (2 dpv)], which was consistent with the findings of a previous study by Walczak et al. (18). After 2 dpv, the AST levels decreased markedly until the last day of life. The high ALT and near-absent AST levels (range, 1–3 U/L) in ASFV-infected pigs during the last 1–2 days of life suggest that liver function was almost completely lost before death. The ALT/AST ratio was used to measure viral hepatitis. The ALT/AST ratio rapidly increased in Group I compared to Group II. Urea levels tended to increase more gradually in Groups I and II, as they had previously (22). However, a sudden increase in creatinine levels during the last 2–3 days of life was observed in Group II pigs, which was not detected in Group I pigs. This suggests that kidney failure was more severe in the later stages [Group II (died at 6–7 dpv)] than in the early stages [Group I (died at 2–5 dpv)] of infection.

Previous studies have reported a sharp increase in the number of macrophages in ASFV target organs, which is associated with the presence of the virus (36). We hypothesized a time-based relationship between the presence of ASFV in various internal organs and the time of death in ASFV-infected pigs. ASFV replicates mainly in mononuclear phagocytic cells in the tonsils or submandibular lymph nodes and then spreads to various internal organs through the blood and lymphatic system (36). In this study, the viral load in the tonsils and lymph nodes did not differ significantly between the two groups. However, it is noteworthy that a significantly higher viral load was detected in the spleens of Group I pigs than in those of Group II pigs (*p* = 0.013), suggesting that the spleen may be more severely affected by ASFV in the early stages of infection. These findings are similar to those reported in a 2014 study of a moderately virulent ASFV strain in Estonia (21). Although the mean values for the lungs, liver, kidney, and colon showed no significant difference, the median value for the lungs was markedly higher in Group II than in Group I. These findings are inconsistent with those of another study on the Estonian strain, which showed that pigs sacrificed at 4 dpi had a higher viral load than those sacrificed at 7 or 10 dpi (21). Differences in the virulence of the ASFV strains may explain the discrepancy in the results between the two studies. Moreover, the tissue samples in our study were collected from pigs that died naturally from the infection, whereas the previous study collected samples from sacrificed pigs (21). Further studies examining the viral loads in organs from a large number of sacrificed pigs per day are required to elucidate the mechanism of ASFV spread in the organs of ASFV-infected pigs.

To determine which internal organs were most affected by ASFV in the early and late stages of infection, we examined the histopathological lesions and viral antigen distributions in various organ tissues of four ASFV-infected pigs [Group I: died at 3 dpv (#54) and 4 dpv (#48); Group II: died at 6 dpv (#60) and 7 dpv (#43)]. Group I pigs had more diverse and severe histopathological lesions in the spleen compared to Group II pigs. Apoptotic or necrotic lymphocytes were more prevalent in the spleen of Group I pigs than in Group II pigs. The severity of histopathological lesions in the spleen was seemingly related to the number of immunolabelled cells. Immunohistochemical analysis revealed fewer ASFV antigen-labeled mononuclear cells or macrophages in the splenic red pulp of pigs that died at 7 dpv than in those that died before 7 dpv, suggesting that the spleen could be more severely damaged within ~1 week after the onset of viremia. These results are consistent with those of a previous study (21). Regarding the submandibular lymph nodes [known to be the first site of virus replication (15)], congestion and hemorrhage gradually decreased with a delay in time to death in individual pigs. In contrast, severe necrotic lesions in the lymphoid follicles were observed more frequently in Group II pigs than in Group I pigs, and apoptosis was only observed in the submandibular lymph nodes of pig #54, which died at 3 dpv. Massive destruction of lymphoid organs and tissues by ASFV infection is attributed to necrotic lymph nodes (41). Similar histopathological lesions were observed in the tonsils and mesenteric and inguinal lymph nodes (Supplementary Table 2). Overall, the results suggest that histopathological lesions of the lymphoid system are more severe in the early stages (Group I) than in the later stages (Group II) of infection. The findings of a previous study support the hypothesis that the apoptosis or necrosis of myelomonocytic cells in spleen tissue peaks at 7 dpi and then decreases at 10 dpi (21).

In the liver, mild-to-moderate and moderate-to-severe lymphocytic infiltrates were present in the sinus of pigs that died in the early (Group I) and late (Group II) stages of infection, respectively. Sinusoidal inflammatory infiltrates are commonly observed in pigs infected with other strains of ASFV (including genotype II in Europe and genotype X in Africa) (17, 42). Notably, pig #54, which died at 3 dpv, showed mild vasculopathy, whereas the other three pigs had severe hemorrhagic lesions. The results were similar to those of a histopathological study of an ASF outbreak at a swine farm in Vietnam (23). The p72 antigen was more pronounced in Group II than in Group I, implying that the liver was more damaged in the later stages than in the early stages of infection. In the lungs, the histopathological changes differed according to the above findings in organs such as the spleen, lymph nodes, and liver. There was no consistent relationship between the lesions in ASFV-infected lungs and the time of death after the onset of viremia. In particular, pig #54 had severe lesions in all categories, including pneumonia, indicating that this pig died from severe pulmonary failure. These results were also evident when immunohistochemistry and polymerase chain reaction were used to evaluate the viral antigen distribution. The effect of ASFV on the lungs depends on the characteristics of the host. Further studies are needed to analyze the host factors that could influence the susceptibility of the lungs to ASFV. In contrast, all ASFV-infected pigs had moderate-to-severe congestion in the liver, which was inconsistent with the findings of a previous case study of pig farms in Vietnam (23). Group II pigs presented more severe kidney lesions than Group I pigs. Hyperemia and bleeding were observed in all pigs, whereas necrosis and interstitial nephritis were only observed in Group II pigs. Although there were no significant differences in immunohistochemical findings or virus titers, the results showed that ASFV could cause serious renal lesions in the later stages of infection. ASFV antigen-labeled cells were detected in the renal tubular epithelium of all pigs in this study, a finding consistent with that in a previous report (23).

Through this pathological study, we aimed to elucidate the course of ASFV infection and the cause of mortality by comparing various organ tissue lesions in pigs that naturally died at 3, 4, 6, and 7 dpv. Our findings provide insights into the mechanism and viral course of systemic infection with ASFV in domestic pigs. However, our study has some limitations with respect to the elucidation of host–virus interactions. As we investigated the pathological findings in only two pigs in each group, no statistical significance could be observed in this study. Consequently, the present findings could not definitively conclude the detailed viral course of ASFV infection in pigs. Additionally, the current data were obtained from pigs that died naturally from ASFV infection; data were not collected from pigs sacrificed daily. Despite close monitoring, the pigs died before reaching the predetermined humane endpoints of the study, which could have affected sample quality. Future studies with large numbers of daily sacrificed pigs could provide a more scientific basis for the pathogenesis of ASFV infection.

## Conclusions

In this study, we investigated the ability of the ASFV strain (VNUA/HY/Vietnam), the first ASFV isolated in Vietnam, to develop pathological processes in infected pigs. Changes in the patterns of complete blood counts and serum biochemical profiles were similar between Group I (pigs that died at 2–5 dpv) and Group II (pigs that died at 6–7 dpv), according to the time of natural death after the onset of viremia. Group I pigs showed more rapid and severe changes in the biochemical parameters of ASFV infection than Group II pigs. Moreover, the mean number of viral copies in the blood of Group I pigs was significantly higher than that in Group II at the onset of viremia, suggesting that individual viral susceptibility was major factor in the timing of death. The viral load of the spleens in Group I was significantly higher than that of the spleens in Group II, indicating that the spleen may be most severely affected during the early stages of ASFV infection. Histopathology and immunohistochemistry also revealed that the spleens and lymph nodes of Group I pigs were more severely affected by ASFV than those of Group II pigs, whereas the liver and kidneys of Group I pigs were less severely affected than those of Group II pigs. These results suggest that the virus could affect the lymphoid system in the early stages of infection and then spread to various internal organs *via* the blood and lymphatic system. However, our study has several limitations with respect to the number of necropsied pigs and the lack of evidence for elucidating the host characteristics according to the time of natural death after the onset of viremia. Therefore, further studies are required to examine pathological lesions in large numbers of sacrificed pigs per day.

## Data availability statement

The original contributions presented in the study are included in the article/Supplementary material, further inquiries can be directed to the corresponding authors.

## Ethics statement

The animal study was reviewed and approved by Institutional Animal Care and Use Committee of the National Institute of Animal Science, the Republic of Korea (approval number: NIAS 2020-463, 2020.2.20.).

## Author contributions

S-IO and HSL made substantial contributions to the conception and design of the work. S-IO, M-SY, BTTN, and BK were responsible for the acquisition and interpretation of pathological data. S-IO, TTHN, VNB, VPL, and HSL were responsible for laboratory analyses. S-IO, S-WY, EK, and T-YH were responsible for the interpretation of the analyzed data. S-IO was a major contributor in writing the manuscript. S-IO, TTHN, and M-SY wrote the original draft. S-IO, HSL, and BK revised the manuscript prior to the submission. All authors read and approved the final manuscript.

## Funding

This work was carried out with the support of the Cooperative Research Program for Agriculture Science and Technology Development (Project title: Analysis and monitoring of clinical and epidemiological features of African swine fever, Project No. PJ01484301), Rural Development Administration, Republic of Korea.

## Conflict of interest

The authors declare that the research was conducted in the absence of any commercial or financial relationships that could be construed as a potential conflict of interest.

## Publisher's note

All claims expressed in this article are solely those of the authors and do not necessarily represent those of their affiliated organizations, or those of the publisher, the editors and the reviewers. Any product that may be evaluated in this article, or claim that may be made by its manufacturer, is not guaranteed or endorsed by the publisher.

## Abbreviations

ASFV: African swine fever virus
ASF: African swine fever
dpi: days post-inoculation
dpv: days post-viremia
HAD_50_/ml: 50% hemadsorbing dose per milliliter
EDTA: ethylene diamine tetra-acetic acid
WBCs: white blood cells
RBCs: red blood cells
AST: aspartate aminotransferase
ALT: alanine aminotransferase.
