# Supplementary material for: Blood parameters and pathological lesions in pigs experimentally infected with Vietnam's first isolated African swine fever virus
Source: Front Vet Sci. 2022 Sep 8;9:978398. doi: 10.3389/fvets.2022.978398 (PMC9495444; doi:10.3389/fvets.2022.978398)
Supplement: Supplementary file 1 [file Data_Sheet_1.docx]

**Supplementary Table 1.** Viral load in the blood sample from each pig with ASFV inoculation.

| Group | Pig case | Death period **^1^** | Viral load in blood depending on days post-viremia (copies/μL) | | | | | | |
| --- | --- | --- | --- | --- | --- | --- | --- | --- | --- |
|  |  |  | 0 | 1 | 2 | 3 | 4 | 5 | 6 |
| Group I | Pig #50 | 2 dpv, 6 dpi | 4.1 × 10^6^ | 4.2 × 10^6^ | - | - | - | - | - |
|  | Pig #54 | 3 dpv, 5 dpi | 5.8 × 10^3^ | 3.3 × 10^6^ | 5.1 × 10^6^ | - | - | - | - |
|  | Pig #48 | 4 dpv, 6 dpi | 1.2 × 10^4^ | 1.3 × 10^6^ | 3.7 × 10^6^ | 1.0 × 10^7^ | - | - | - |
|  | Pig #58 | 4 dpv, 6 dpi | 5.9 × 10^3^ | 3.8 × 10^6^ | 1.0 × 10^7^ | 8.8 × 10^6^ | - | - | - |
|  | Pig #40 | 5 dpv, 7 dpi | 6.0 × 10^4^ | 3.7 × 10^6^ | 6.3 × 10^6^ | 1.6 × 10^7^ | 1.1 × 10^7^ | - | - |
| Group II | Pig #56 | 6 dpv, 8 dpi | 1.2 × 10^1^ | 9.6 × 10^2^ | 1.4 × 10^5^ | 9.2 × 10^5^ | 5.9 × 10^6^ | 8.6 × 10^6^ | - |
|  | Pig #60 | 6 dpv, 8 dpi | 3.3 × 10^3^ | 2.6 × 10^6^ | 1.4 × 10^6^ | 3.6 × 10^6^ | 3.8 × 10^6^ | 6.3 × 10^6^ | - |
|  | Pig #67 | 6 dpv, 8 dpi | 4.5 × 10^1^ | 4.6 × 10^4^ | 2.2 × 10^6^ | 7.8 × 10^6^ | 1.1 × 10^7^ | 6.0 × 10^6^ | - |
|  | Pig #59 | 7 dpv, 8 dpi | 1.1 × 10^2^ | 1.4 × 10^6^ | 3.1 × 10^6^ | 6.8 × 10^6^ | 4.2 × 10^6^ | 4.2 × 10^6^ | 8.1 × 10^6^ |
|  | Pig #43 | 7 dpv, 8 dpi | 1.7 × 10^1^ | 1.1 × 10^3^ | 4.6 × 10^6^ | 2.4 × 10^6^ | 1.1 × 10^7^ | 1.1 × 10^7^ | 1.3 × 10^7^ |

^1^ dpv, days post-viremia; dpi, days post-inoculation

**Supplementary Table 2.** Histopathological spleen, submandibular lymph node, liver, lung, kidney, mesenteric and inguinal lymph node, tonsil, colon, and heart lesions in pigs experimentally infected with African swine fever virus.

| **Organ** | **Histopathological findings** | **Score ^1^** | | | | |
| --- | --- | --- | --- | --- | --- | --- |
|  |  | **Group I** | |  | **Group II** | |
|  |  | **Pig #54** | **Pig #48** |  | **Pig #60** | **Pig #43** |
| Spleen | Apoptosis | ++ | ++ |  | + | + |
|  | Lymphoid depletion | +++ | ++ |  | + | ++ |
|  | Necrosis | ++ | +++ |  | + | + |
|  | Vascular damage | ++ | +++ |  | +++ | + |
| Submandibular lymph nodes | Congestion/hemorrhage | +++ | ++ |  | + | + |
|  | Apoptosis | + | – |  | – | – |
|  | Necrosis | + | ++ |  | +++ | +++ |
|  | Lymphocyte hyperplasia | + | +++ |  | ++ | ++ |
| Liver | Congestion/hemorrhage | + | +++ |  | +++ | +++ |
|  | Apoptosis | ++ | + |  | ++ | + |
|  | Necrosis | – | + |  | + | + |
|  | Sinusoidal inflammatory infiltrates | + | ++ |  | ++ | +++ |
| Lungs | Congestion | +++ | ++ |  | +++ | ++ |
|  | Hemorrhage | +++ | – |  | +++ | – |
|  | Pulmonary edema | +++ | + |  | ++ | + |
|  | Interstitial pneumonia | +++ | + |  | ++ | + |
| Kidneys | Congestion | ++ | + |  | +++ | +++ |
|  | Hemorrhage | ++ | ++ |  | +++ | + |
|  | Necrosis | – | – |  | + | – |
|  | Interstitial nephritis | – | – |  | +++ | ++ |
| Mesenteric lymph nodes | Congestion/hemorrhage | + | ++ |  | +++ | +++ |
|  | Apoptosis | + | + |  | – | + |
|  | Necrosis | + | ++ |  | ++ | ++ |
|  | Lymphocyte hyperplasia | + | +++ |  | +++ | ++ |
| Inguinal lymph nodes | Congestion/hemorrhage | + | + |  | ++ | ++ |
|  | Apoptosis | – | + |  | – | – |
|  | Necrosis | + | +++ |  | +++ | +++ |
|  | Lymphocyte hyperplasia | – | + |  | + | +++ |
| Tonsils | Congestion/hemorrhage | – | ++ |  | + | – |
|  | Apoptosis | +++ | +++ |  | + | + |
|  | Necrosis | + | +++ |  | +++ | +++ |
|  | Crypt abscessation | + | ++ |  | ++ | ++ |
| Colon | Hemorrhage | NT | – |  | ++ | + |
| Heart | Congestion | – | + |  | + | – |

^1^ Scored according to previously published guidelines (Galindo-Cardiel et al., 2013; Walczak et al., 2021). No lesion (–), mild (+), moderate (++), severe (+++), not tested (NT).

**Supplementary Table 3.** Immunohistochemical analysis of mesenteric and inguinal lymph nodes, tonsils, colon, and heart from pigs experimentally infected with African swine fever virus

| **Organ** | **Score** ^1^ | | | | |
| --- | --- | --- | --- | --- | --- |
|  | **Group I** | |  | **Group II** | |
|  | **Pig #54** | **Pig #48** |  | **Pig #60** | **Pig #43** |
| Mesenteric lymph nodes | +++ | ++++ |  | ++++ | ++++ |
| Inguinal lymph nodes | ++ | ++ |  | ++++ | +++++ |
| Tonsils | +++ | ++++ |  | ++++ | ++ |
| Colon | ++ | +++ |  | +++ | +++ |
| Heart | – | + |  | + | + |

^1^ Scored according to the proportion of positively stained mononuclear cells or macrophages in three fields under ×400 magnification. No positive cells (–), 1–10 positive cells (+), 11–20 positive cells (++), 21–30 positive cells (+++), 31–40 positive cells (++++), ≥41 positive cells (+++++).
